# Awareness, Knowledge, and Acceptance of Living Wills Among Chinese Cancer Patients: A Cross‐Sectional Study

**DOI:** 10.1002/hcs2.70027

**Published:** 2025-09-02

**Authors:** Longxia Hu, Jiaqing Wang, Deying Hu, Yilan Liu, Yanhong Han, Wenbin Liu, Fen Teng, Xiaoping Ding, Yi Dai, Lihong Bao, Hongling Zhang, Qingzhou Cheng, Yun Yang, Tingting Jiao, Fei Luo, Yali Yang, Zixi Wang, Xiaoping Yang, Shengli Yang

**Affiliations:** ^1^ Cancer Center, Union Hospital, Tongji Medical College Huazhong University of Science and Technology Wuhan China; ^2^ Department of Nursing, Union Hospital, Tongji Medical College Huazhong University of Science and Technology Wuhan China; ^3^ College of Medicine and Health Science Wuhan Polytechnic University Wuhan China; ^4^ Department of Endocrinology, Union Hospital, Tongji Medical College Huazhong University of Science and Technology Wuhan China

**Keywords:** attitude, knowledge, living will, malignant tumor, palliative care

## Abstract

**Background:**

Given the suboptimal quality of end‐of‐life care among patients with cancer in China, promoting living wills is critical in this population. Living wills ensure that individuals can receive the medical care they desire during the terminal phase of an illness, maintain their dignity, and ultimately achieve a good death. However, current awareness and attitudes about living wills among Chinese patients with cancer remain unclear. We administered a questionnaire survey on living wills to patients with malignant tumors to assess their most important needs and increase understanding about living wills.

**Methods:**

In this cross‐sectional study using convenience sampling, inpatients with malignant tumors in Wuhan completed our questionnaire between July 2020 and June 2021. We collected patients' sociodemographic characteristics and details regarding their knowledge and attitudes about living wills.

**Results:**

Among 213 patients with malignant tumors, 114 (53.52%) had heard of living wills; 125 (58.69%) expressed their willingness to sign the “Five Wishes” living will document after learning about it through the questionnaire. Patients with malignant tumors had a high level of desire for the following living will items: the lives of family and friends return to normal as soon as possible after their death, maintaining personal hygiene and dignity, and remaining pain‐free. The knowledge level of patients with malignant tumors was related to their educational level (*p* < 0.05) and self‐care ability (*p* < 0.05).

**Conclusions:**

Patients with malignant tumors have a high need for comfort, cleanliness, and pain relief in the terminal stages. Patients with a higher level of education and those with poorer self‐care ability had greater knowledge and acceptance of living wills. Promotion can first be targeted toward more highly educated patients and can then be gradually expanded to other groups.

## Graphical abstract

Patients with higher educational levels or with poorer self‐care ability had greater knowledge and acceptance of living wills. Cancer patients have greater need for comfort, dignity, and pain relief in the terminal stages. Promotion can initially target more highly educated patients and can then be gradually expanded.

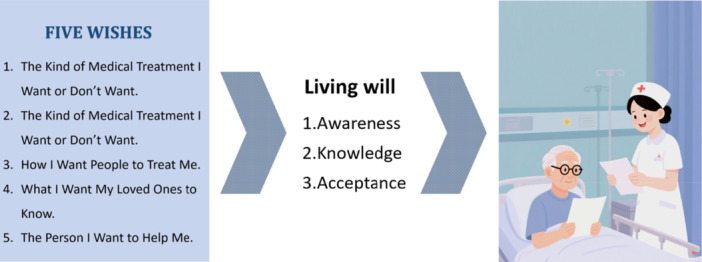

**Abbreviation**
ADL: activities of daily living

## 1 Introduction

As a leading cause of death and an important barrier to increasing life expectancy worldwide, cancer caused nearly 10 million deaths in 2020, according to statistics by GLOBOCAN [1]. China has a large population base, and the incidence of cancer in China is also high; therefore, Chinese patients with malignant tumors form a large group. In February 2022, the latest national cancer statistics released from the National Cancer Center of China showed there were 380 million people diagnosed with malignant tumors in China [2].

With ongoing development of medical technology, increasingly more patients with tumors have longer life expectancy owing to advanced life support technologies and emerging anticancer therapies [3]. However, the quality of death among patients with tumors is not optimistic [4]. The quality of death and dying refers to the degree of similarity between an individual's preference regarding the manner and time of their death, and their actual death as observed by others [5]. At present, the quality of death in China ranks 53rd among 80 countries and regions worldwide [6]. To some extent, this ranking reflects an experience of patients with cancer at the end of their life that is inconsistent with their own expectations of death. Thus, the quality of death in these patients should be improved. Patients in the terminal and dying stages undergo various types of treatment that may limit their ability to express their wishes. This causes the patient emotional pain and increases psychological pressure on the family [7], as well as a heavy economic burden [8]. Ultimately, many patients who could have avoided painful treatments die in pain and feeling regret. In 2024, the National Health Commission issued the *Guidance on Strengthening the Construction of Palliative Care Service System*, which proposed the establishment and improvement of a palliative care service network to enhance quality at the end of life for terminally ill patients.

A living will is a document that guarantees an individual can obtain the medical care they want in the terminal stage of an illness when they cannot express their wishes [9]. The document is signed when a person is healthy or conscious and is used to indicate whether certain types of medical and nursing care should be used in the terminal or dying stages of an incurable illness/disease [9]. A living will guarantees patient autonomy and dignity to the greatest extent and improves the quality of death [10]. A living will document usually includes multiple‐choice questions, and different countries have their own unique living will text. Currently, the use of living wills in China is limited, and many people have no knowledge about living wills. Living wills have not been legally guaranteed in China, which also hinders people's interest in registering a living will.

Few studies have concentrated on knowledge and attitudes about living wills among Chinese patients with malignant tumors. In our previous qualitative research, the topic of living wills was found to be relatively sensitive for patients with malignant tumors [11]. However, it is important to promote living wills among these patients. In this study, we used items from the “Five Wishes” living will document to describe the current status of knowledge and attitudes about living wills among Chinese patients with malignant tumors and to explore their influencing factors.

## 2 Methods

### 2.1 Design

We conducted a cross‐sectional study to investigate the attitudes of patients with malignant tumors toward living wills by developing and validating our proposed scale, the “Questionnaire on Knowledge, Attitudes, and Wishes Regarding Living Wills Among Patients with Malignant Tumor.” Because the main part of the questionnaire included original text from the “Five Wishes” living will document with only minor modifications and mainly multiple‐choice items, we also used expert consultation to conduct validity verification and ensure the rationality and effectiveness of the questionnaire design. This questionnaire was deemed suitable for conscious patients with cancer over 18 years old.

### 2.2 Measurement

We developed a self‐administered questionnaire on knowledge about and attitudes toward living wills for patients with malignant tumors. The questionnaire was developed on the basis of the “Five Wishes,” a literature review, and two rounds of expert consultation (Supporting information).

The questionnaire was divided into four parts. Part I comprised general information, including sex, age, marital status, education, self‐care ability, family members, residence, and religious beliefs. Part II included two questions. Because patients with malignant tumors are more sensitive to discussions about death and dying, we first asked, “Are you willing to talk about dying” To understand respondents' knowledge about living wills, we then asked, “Have you heard of a living will”

Part III contained six items in total, including 10 sub‐items. This part comprised specific content in the original “Five Wishes” living will document and all its options, as follows: “What medical services do I want/not want; I want to use/not use life support treatment; How I want others to treat me; What I want my family and friends to know; Who I want to help me.” To further understand the reasons why respondents wanted/did not want life support treatment at the end of their life (according to their response to the second item, I want to use/not use life support treatment), we added a sub‐item for those who stated that they wanted life support treatment. These respondents were further asked to give the reasons for their responses.

Part IV included only one question. To understand respondents' acceptance of the “Five Wishes” living will document, at the end of the questionnaire, we asked, “Are you willing to sign the ‘Five Wishes’ living will document.”

### 2.3 Participants

A convenience sampling method was used to randomly select hospitalized patients with malignant tumors at a tertiary hospital in Wuhan between July 2022 and June 2023 as research participants. Inclusion criteria were: (1) Age ≥ 18 years; (2) Definite diagnosis of malignant tumor; (3) No mental impairment or intellectual deficiency; (4) Able to complete the questionnaire, and willing to cooperate with the investigation. The exclusion criteria were: (1) Unable to understand the content of the questionnaire; (2) Patients who preferred to avoid topics related to dying and death. The sample size was calculated as 5–10 times the number of questions, considering a 10% loss rate, that is, (13 × [5–10]) × (1 + 10%); the sample size was calculated to be 72–143 patients.

### 2.4 Data Collection and Quality Assurance

The questionnaire was completed by eligible patients who met the inclusion criteria via scanning of a QR code. To ensure the quality of completed surveys, the questionnaire was administered one‐to‐one. During the survey process, the researcher explained the method for completing the questionnaire to participants, instructing them to respond according to their true wishes. A total of 215 patients with malignant tumors completed the survey; two invalid questionnaires were removed because the patient only completed some items themselves and had family members complete the remaining items. Thus, a total of 213 valid surveys were collected, with an effective response rate of 99.07%.

### 2.5 Data Analysis

We used IBM SPSS 26.0 for data analysis (IBM Corp., Armonk, NY, USA), and descriptive statistics were used for count data. Comparisons between two or more sample rates (or constituent ratios) were performed using the chi‐square test, and the interdependent relationship between variables was analyzed using logistic regression. All statistical tests were two‐sided, with test level *α* = 0.05.

## 3 Results

### 3.1 Demographics

Among the 213 participants who submitted a valid questionnaire, the age range was 25–83 (49.24 ± 13.93) years; 124 were men (58.22%) and 89 (41.78%) were women. As for self‐care ability, 194 (91.08%) participants had full self‐care ability, and 19 (8.92%) were unable to take care of themselves completely. Detailed demographic information can be found in Table 1.

**Table 1:** Demographic information (*N* = 213).

General information	*n*	%
Age (years)		
20–39	40	18.7
40–59	124	58.3
≥ 60	49	23.0
Gender		
Male	124	58.3
Female	89	41.7
Marital status		
Married	203	95.3
Unmarried	10	4.7
Education		
Primary school or below	19	8.9
Junior high school	57	26.8
Senior high school	72	33.8
University	65	30.5
Self‐care ability (Barthel index of ADL)		
Adequate	190	89.2
Inadequate	23	10.8
Family members		
Spouse	191	89.7
Children	187	87.8
Parents	113	53.1
Brothers and sisters	156	73.2
Residence		
City	141	66.2
Town	72	33.8
Religious faith		
Yes	17	8.0
No	196	92.0

Abbreviation: ADL, activities of daily living.

### 3.2 Knowledge and Acceptance of Living Wills in Patients With Malignant Tumors

The survey results showed that among the 213 patients with malignant tumors, 114 (53.52%) had heard of a living will, and 125 (58.69%) felt they were knowledgeable about and willing to sign a living will after completing the questionnaire.

### 3.3 Analysis of Factors Related to Knowledge About Living Wills in Patients With Malignant Tumor

Statistical analysis of the general data and knowledge level of patients with malignant tumors showed that educational level (*χ* ^2^ = 222.64, *p* < 0.05) and self‐care ability (*χ* ^2^ = 4.310, *p* < 0.05) were significantly related to their knowledge about living wills. These levels were closely related. Patients with better knowledge about living wills included a higher proportion with higher education levels (college or junior college: 41/65, 63.08%; senior high school or polytechnic school: 33/72, 45.83%; primary school education or below: 7/19, 36.84%). Patients with better knowledge about living wills included a higher proportion with poorer self‐care ability (97/190, 51.05% vs. 17/23, 73.91%). Age, marital status, habitual residence, and religious beliefs were not significantly correlated with knowledge about living wills in patients with malignant tumors (Table 2).

**Table 2:** Analysis of factors related to knowledge levels about living wills in patients with malignant tumor.

General information	*n*	Have heard about it, *n* (%)	Have never heard of it, *n* (%)	*χ* ^2^	*p*
Age (years)				0.034	0.983
20–39	40	21 (52.50)	19 (47.50)		
40–59	124	67 (54.03)	57 (45.97)		
≥ 60	49	26 (53.06)	23 (46.94)		
Gender				0.538	0.463
Male	124	69 (55.65)	55 (44.35)		
Female	89	45 (50.56)	44 (49.44)		
Marital status				0.063	0.802
Married	203	108 (53.20)	95 (46.80)		
Unmarried	10	6 (60.00)	4 (40.00)		
Education				222.64	< 0.001*
Primary school or below	19	7 (36.88)	12 (63.16)		
Junior high school	57	33 (57.89)	24 (42.11)		
Senior high school or polytechnic school	72	33 (45.83)	39 (54.17)		
Junior college, university or above	65	41 (63.08)	24 (36.92)		
Self‐care ability				4.31	0.038*
Adequate	190	97 (51.05)	93 (48.95)		
inadequate	23	17 (73.91)	6 (26.09)		
Family members				0.04	0.998
Spouse	191	101 (52.88)	90 (47.12)		
Children	187	99 (52.94)	88 (47.06)		
Parents	113	61 (53.98)	52 (46.02)		
Brothers and sisters	156	83 (53.21)	73 (46.79)		
Residence				1.054	0.305
City	141	79 (56.03)	62 (43.97)		
Town	72	35 (48.61)	37 (51.39)		
Religious faith				0.002	0.960
Yes	17	9 (52.94)	8 (47.06)		
No	196	105 (53.57)	91 (46.43)		

* Statistically significant.

### 3.4 Analysis of Factors Related to Whether Patients With Malignant Tumors Were Willing to Sign a Living Will

Statistical analysis of the general knowledge and attitudes among patients with malignant tumors showed that educational level (*χ* ^2^ = 12.054, *p* < 0.05) was closely related to patients' attitudes regarding willingness to sign a living will. Among patients with malignant tumors who were willing to sign a living will, 73.85% had a college degree or above, 58.33% had a high school or technical secondary school education, 49.12% had a junior high school education, and 36.84% had a primary school education or below (Table 3).

**Table 3:** Analysis of factors related to whether patients with malignant tumors were willing to sign a living will.

General information	*n*	Willing to, *n* (%)	Not willing to, *n* (%)	*χ* ^2^	*p*
Age				1.597	0.450
20–39	40	20 (50.00)	20 (50.00)		
40–59	124	76 (61.29)	48 (38.71)		
≥ 60	49	29 (59.18)	20 (40.82)		
Gender				0.396	0.529
Male	124	75 (60.48)	49 (39.52)		
Female	89	50 (56.18)	39 (43.82)		
Marital status				0.007	0.931
Married	203	119 (58.62)	84 (41.38)		
Unmarried	10	6 (60.00)	4 (40.00)		
Education				12.054	0.007*
Primary school or below	19	7 (36.84)	12 (63.16)		
Junior high school	57	28 (49.12)	29 (50.88)		
Senior high school or polytechnic school	72	42 (58.33)	30 (41.67)		
College, university or above	65	48 (73.85)	17 (26.15)		
Self‐care ability				0.451	0.502
Adequate	190	113 (59.47)	77 (40.53)		
inadequate	23	12 (52.17)	11 (47.83)		
Family members				0.509	0.917
Spouse	191	114 (59.69)	77 (40.53)		
Children	187	108 (57.75)	79 (42.25)		
Parents	113	63 (55.75)	50 (44.25)		
Brothers and sisters	156	89 (57.05)	67 (42.95)		
Residence				0.136	0.712
City	141	84 (59.57)	57 (40.43)		
Town	72	41 (56.94)	31 (43.06)		
Religious faith				0.002	0.966
Yes	17	8 (47.06)	9 (52.94)		
No	196	117 (59.69)	79 (40.31)		

* Statistically significant (*p* < 0.05).

### 3.5 Multivariate Analysis of Whether Patients With Malignant Tumors Were Willing to Sign a Living Will

#### 3.5.1 Variable Assignment

Binary logistic regression was used to analyze the factors influencing whether patients with malignant tumors were willing to sign a living will. The general data variables assigned are shown in Table 4.

**Table 4:** Related factors and assignment description of whether patients with malignant tumors are willing to sign a living will.

Factors	Variable name	Assignment description
Education	*X*_1_	1 = Primary school and below, 2 = Junior high school, 3 = High school or technical secondary school, 4 = College and above
Self‐care ability	*X*_*2*_	0 = inadequate, 1 = adequate
Whether willing to sign	*Y*	0 = no, 1 = yes

#### 3.5.2 Logistic Regression Analysis of Whether Patients With Malignant Tumors Were Willing to Sign a Living Will

The dependent variable (*Y)* referred to whether patients with malignant tumors were willing to sign a living will, where “1” indicated willingness to sign and “0” indicated unwillingness to sign. On the basis of the chi‐square test, we conducted logistic regression analysis using educational level and self‐care ability as independent variables (*X*). The results showed that education level was significant in the regression equation (*χ* ^2^ = 12.302, *p* = 0.006). The higher the patient's education level, the more willing they were to sign a living will (Table 5).

**Table 5:** Logistic regression analysis of whether patients with malignant tumors are willing to sign a living will.

Variable (educational level)	*B*	SE	*df*	*p*	OR	95% CI
Primary school			3	0.009*	1	
Junior high school	0.504	0.544	1	0.355	1.655	0.569–4.811
High school or polytechnic school	0.857	0.532	1	0.100	2.400	0.846–6.812
College and above	1.577	0.533	1	0.004*	4.840	1.637–14.309
Constant	−0.539	0.476	1	0.257	0.538	

Abbreviations: CI, confidence interval; *df*, degrees of freedom; OR, odds ratio; SE, standard error.

* Statistically significant (*p* < 0.05).

## 4 Discussion

### 4.1 Knowledge About Living Wills in Patients With Malignant Tumors

In this survey, 53.52% of patients with malignant tumors had heard of the living will, which is higher than the proportion among the general Chinese population (38.3%) [12] and far higher than the result of a population‐based cross‐sectional survey conducted by telephone in Hong Kong, China (14.3%) [13].

Malignant tumors usually indicate a disease that cannot be completely cured, posing a great threat to the health and life of the patient. Once a patient is diagnosed with a malignant tumor, this often means that death will occur earlier than among individuals without this type of diagnosis. This prompts some patients with malignant tumors to think more than usual about topics related to death and dying, which are topics addressed in the living will.

Compared with other departments, it is more common for medical staff in the oncology department to participate in educational activities related to hospice care, death education, and promotion of living wills. There are also more studies on the implementation of palliative care, death education, and promotion of living wills among patients with malignant tumors. For those patients with malignant tumors who have no obvious resistance to a living will, the possibility of receiving education and acquiring knowledge about living wills is relatively high, and related concepts are more widely accepted.

Owing to the long course of disease and the need for treatment, patients with malignant tumors are often hospitalized repeatedly. Compared with general patient populations, it is more common for patients with cancer to become critically ill or die as the result of disease progression. These reasons prompt patients with malignant tumors to pay greater attention to and try to understand end‐of‐life events to some extent, such that the knowledge level of these patients is higher than that of the general population.

### 4.2 Factors of Knowledge About Living Wills in Patients With Malignant Tumors

Our survey showed that educational level (*χ* ^2^ = 222.64, *p* < 0.05) and self‐care ability (*χ* ^2^ = 4.31, *p* < 0.05) were closely related to knowledge about living wills in patients with malignant tumors. Patients with higher educational levels had greater knowledge levels about living wills than those with low educational levels. Similarly, another study in China found that patients with a college‐level education or more had greater preference for making advance directives [14]. Another study also showed that participants with higher educational attainment were more willing to discuss the option of a living will [15]. People with a high level of education often have more channels via which to receive information and greater comprehension abilities than those with a low level of education; also, their ability to view problems correctly and rationally is also greater than that of patients with a low education level. Therefore, knowledge levels regarding living wills is lower among patients with a lower level of education.

Patients who could not completely care for themselves were more knowledgeable about living wills than those with complete self‐care ability. Patients who cannot fully take care of themselves may have more severe symptoms owing to their disease, or the disease course may be closer to the end stage [16]. Therefore, these patients may have engaged in a certain degree of inner reflection or have a certain level of psychological preparation for death‐related matters. By understanding the experience of other terminal patients, patients may anticipate situations they may face in the future, and may have a clearer understanding of what they want at their death time. Some patients will also state their wishes for after their death so that their family clearly understands what the patient wants.

### 4.3 Living Will Items Frequently Chosen by Patients With Malignant Tumors

Among respondents, 84% chose the item, “I hope my family and friends can return to normal life as soon as possible after my death.” The reasons given for choosing this statement were as follows: (1) Family members and friends are the closest to the patient's heart, so patients have the most concern for them. (2) During the patient's hospitalization and while undergoing various treatments and examinations, the patient's family took care of them with great love and spent much time being together with them. (3) The patient's death will deal a psychological blow to and increase pressure on family members and people who care about them, and the patient feels guilty about this [17]. (4) During the treatment process, the economic cost is very high, with high expenses that must be paid for by the family; therefore, some patients with malignant tumors do not wish to place a greater economic burden on their family with extended treatments. For these reasons, patients hope that their family and friends can return to normal life as soon as possible after their death. This statement also reflects the need for emotional expression among patients with malignant tumors. Therefore, in clinical health education for patients' families, it is necessary to encourage interactive expression of such emotions to help avoid regret owing to not voicing true feelings in the hearts of patients and their families.

Among our survey respondents, 67.6% and 61.5% chose the items, “I want my bed to be dry and clean, and if it is contaminated, I want it to be changed as quickly as possible” and “I want my body to be clean and odorless at all times,” respectively. These items indicate that some patients want to be kept clean and comfortable when they die to maintain their dignity. In clinical practice, medical staff should improve observation and communication with patients to determine patients' needs for cleanliness and comfort in a timely and active manner, respect and meet the basic needs of patients, and help patients feel comfortable and dignified.

Among patients with malignant tumors, 65% chose the item, “I don't want pain, I hope they give me enough medicine for pain relief.” Patients with malignant tumors have a strong desire to have no pain in the terminal stage of their disease, which may be related to their knowledge that patients may experience cancer pain in the terminal stages. The National Comprehensive Cancer Network clinical practice guidelines in oncology: adult cancer pain in the United States point out that cancer pain is not only one of the most feared symptoms among patients, this type of pain also seriously affects patients' daily activities, interests, interaction with family and friends, and overall quality of life [18]. Therefore, many patients wish to have no cancer pain. Greater attention is needed to pain management among patients with malignant tumors in daily nursing work, and pain relief should be prioritized and minimized to promote patient comfort. Experiencing pain can lead to psychological distress and depression, and limited physical functioning owing to pain can even lead to suicidal thoughts [19]. For this reason, the psychological care of patients must be improved to reduce the occurrence of anxiety, depression, and suicidal thoughts or behavior [20].

### 4.4 Living Will Items Less Frequently Chosen by Patients With Malignant Tumors

Only 4.69% of our survey respondents chose the item, “I would like to have a certain religious ceremony conducted at the end of my life.” This low proportion is probably because most patients surveyed stated that they had no religious beliefs.

Only 14% of patients selected the items “Accept as many volunteer services as possible” and “Don't be disturbed by volunteers at any time.” Patients with malignant tumors want to be accompanied at the end of their life. Dying patients are usually accompanied by relatives or people with whom they have an important relationship. Patients hope to spend their limited remaining time with relatives or important friends as much as possible. Volunteers are relatively unimportant to patients; therefore, few patients surveyed felt that items related to volunteers were important. Research has confirmed that volunteers can establish close interpersonal relationships with patients and provide them with personalized care, and may play an important role in discussions about advance directives and care needs [21]. Therefore, volunteer services can be appropriately introduced in palliative care work to help patients better understand and benefit from this type of support.

Among survey respondents, only 16% stated, “There are pictures or photos that I like hanging by my bedside.” This indicated that having pictures or photos to look at is not very meaningful to these patients. Tishelman et al. found that encouraging patients who are receiving palliative care to bring personal items into the palliative care ward and arrange them according to their preferences can help them to maintain continuity regarding their physical environment and social functioning before admission. Incorporating personal items into patients' physical environment on the ward has positive effects on the psychological well‐being, social interactions, and autonomy of patients in the terminal stages [22]. Therefore, in clinical practice, encouraging terminally ill patients to bring small, cherished items to decorate their rooms is important.

## 5 Strengths and Limitations

For the first time, the “Five Wishes” living will document was presented to patients with malignant tumors in the form of a questionnaire, allowing patients to think about their choices when facing dying and their own death, while in a relatively relaxed atmosphere. Additionally, our questionnaire survey revealed important points regarding the difficulties in discussing death and dying face‐to‐face with patients who have malignant tumors, which provide a theoretical basis and reference for the promotion and implementation of living wills among these patients.

This study also has some limitations. Owing to the sensitive topic of living wills and the physical condition of survey respondents, the implementation of the present research was relatively difficult. In terms of sample selection, only patients hospitalized in one large tertiary hospital in Wuhan were selected as research participants, which may not be representative of the overall situation regarding knowledge and attitudes among patients with malignant tumors toward advance directives. Knowledge and attitudes about living wills among patients with cancer is affected by their educational level. Patients with low education levels have insufficient understanding and ability to express their wishes regarding living wills; there is insufficient in‐depth research on knowledge and attitudes about living wills in this patient group.

We used a questionnaire format to guide patients to think about their dying process and death‐related matters and to give them some direction and goals to reflect on. In promoting living wills, a similar survey can be used as a starting point to gradually introduce death education as well as new ideas and planning options for patients with malignant tumors.

## 6 Conclusion

Patients with malignant tumors are sensitive to the topic of death and dying, so it may be difficult to promote living wills among these patients. Our patients with high educational levels showed greater knowledge and acceptance of living wills than did patients with low educational levels. The targets of promotion can start with more highly educated patients and those with a good doctor–patient relationship, and then can be gradually expanded according to the acceptance of patients and their families. When talking about topics related to a living will, the opportunity should be taken to share relevant knowledge about dying with dignity through the use of a living will, while also fully considering the attitudes and feelings of family members. In clinical work, patients with malignant tumors have a high need for physical cleanliness, comfort, dignity, and pain relief. Greater attention is needed regarding patients' wishes in these areas to proactively meet their needs and help them maintain comfort and dignity to improve their quality of life.

## Author Contributions

**Longxia Hu:** writing – original draft (equal). **Jiaqing Wang:** writing – original draft (equal). **Deying Hu:** conceptualization (lead). **Yilan Liu:** conceptualization (equal). **Yanhong Han:** conceptualization (equal). **Wenbin Liu:** writing – review and editing (supporting) & resources (equal). **Fen Teng:** formal analysis (equal). **Xiaoping Ding:** methodology (equal). **Yi Dai:** methodology (equal). **Lihong Bao:** methodology (equal). **Hongling Zhang:** validation (equal). **Qingzhou Cheng:** data curation (equal). **Yun Yang:** investigation (equal). **Tingting Jiao:** investigation (equal). **Fei Luo:** investigation (equal). **Yali Yang:** investigation (equal). **Zixi Wang:** investigation (equal). **Xiaoping Yang:** investigation (equal). **Shengli Yang:** resources (equal).

## Ethics Statement

The study was approved by the Ethics Committee of Tongji Medical College, Huazhong University of Science and Technology (NO.:IORG0003571) and informed consent was taken from all individual participants.

## Consent

Informed consent was obtained from all subjects or their families.

## Conflicts of Interest

The authors declare no conflicts of interest.

## Supporting information


**Supporting Information:** Questionnaire on Cognition, Attitude and Wish of Living Will in Patients with Malignant Tumors.

## Data Availability

The data that support the findings of this study are available from the corresponding author upon reasonable request.
