# Supplementary material for: Awareness, Knowledge, and Acceptance of Living Wills Among Chinese Cancer Patients: A Cross‐Sectional Study
Source: Health Care Sci. 2025 Sep 2;4(5):319–27. doi: 10.1002/hcs2.70027 (PMC12574422; doi:10.1002/hcs2.70027)
Supplement: Supplementary file 1 — Supporting Information: Questionnaire on Cognition, Attitude and Wish of Living Will in Patients with Malignant Tumors. [file HCS2-4-319-s001.pdf]

Questionnaire on Cognition, Attitude and Wish of Living Will in Patients with Malignant Tumors

Hello! In order to improve the quality of medical care and provide better care for patients, we are currently investigating the attitudes and wishes of tumor patients and their families regarding living wills. Please fill out the questionnaire above truthfully. Living wills aim to ensure that individuals can receive the medical care they want even when they are unable to express their wishes during the terminal phase of an illness. Regardless of whether one is healthy or ill, everyone will eventually reach the end of life. What wishes would you like to fulfill at the end of your life? This survey will not affect your current medical care. Any personal information involved will be kept strictly confidential, so please feel free to complete it. Thank you for your cooperation! We wish you a pleasant day!

Part Ⅰ

1.Identity *

○ Patient

○ Patient’s family

2.Gender *

○ Male

○ Female

3.Age *

____

4.Marital status *

○ Married

○ Unmarried

○ Divorce

5.Educational level *

○ Primary school or below

○ Junior high school
○ Senior high school
○ Undergraduate degree
○ Graduate degree

6.Self-care ability *
○ Wholly
○ Partly
○ None

7.What family members do you have?*（Multiple-choice question）
□ Spouse
□ Child
□ Parents
□ Brothers and sisters

8.Permanent residence *
○ City
○ Rural area
○ Town

9.Religious belief *
○ None
○ Buddhism
○ Christianity
○ Islam
○ Others ____________

Part Ⅱ

10.Are you willing to talk about your dying?*
○ Yes
○ No

11.Have you ever heard about living wills?*
○ Yes, I have.

○ No, I haven’t.

Part Ⅲ

Wish 1 My Wish For The Kind Of Medical Treatment I Want Or Don’t Want.

12.I believe that my life is precious and I deserve to be treated with dignity. When the time comes that I am very sick and am not able to speak for myself, I want the following wishes, and any other directions I have given to my Health Care Agent, to be respected and followed. * (multiple-choice question)

□ I do not want to be in pain. I want my doctor to give me enough medicine to relievemy pain.

□ If I show signs of nausea, spasm, convulsion, delirium, fear or hallucination, I want my care givers to do whatever they can to help me.

□ I do not want any treatment or examination that would increase my pain (such as radiotherapy, chemotherapy, surgical exploration, etc.), even if the doctors and nurses think it may be helpful in clarifying the diagnosis and improving my symptoms.

□ I hope that my privacy will be fully protected during treatment and care.

□ I want my body to be clean and odorless all the time.

□ I wish to have personal care like shaving, nail clipping, hair brushing, and teeth brushing.

□ I wish my bed linens to be kept clean, and for them to be changed as soon as they can be if they have been soiled.

□ I wish the food and water provided for me to be kept clean and warm at all times.

□ I wish to donate my organs and tissues of useful as permitted by law.

□ Others ____________

Wish 2 My Wish For The Kind Of Medical Treatment I Want Or Don’t Want

13.Would you give up life-support treatment at the end of life? *

○ Yes (If you answered yes, please skip to Question 14)

○ No (If you answered no, please skip to Question 15)

14.The reason for you to give up life-support treatment:（multiple-choice question）

□ It may make me suffer more.

□ I wish to die with dignity.

□ Huge cost

□ Don’t want drag family down

□ Others ____________

15.The reason for you not to give up life-support treatment:（multiple-choice question）
□ To live as long as possible, even without any quality of life or awareness.
□ Be afraid of death
□ Whether to receive life-support treatment is decided by my family, and I cannot change it.
□ Others ____________

16.I know that life-support treatment is the only way to keep me alive. But when my survival is of no quality and life-support treatment can only prolong my dying process, I will carefully consider whether to use it. Note! When I request not to use life-support treatment, it only includes: * (multiple-choice question)
□ cardiopulmonary resuscitation (CPR)
□ ventilator
□ tube feeding
□ blood transfusions
□ expensive antibiotics

Close To Death
17.If my doctor and another health care professional both decide that I am likely to die within a short period of time(Survival no more than 6 months), and life-support treatment would only delay the moment of my death (choose one of the following): (*)
○ 1 I want to have life-support treatment.
○ 2 I do not want life-support treatment. If it has been started, I want it stopped.
○ 3 I want to have life-support treatment if my doctor believes it could help. But I want my doctor to stop giving me life-support treatment if it is not helping my health condition or symptoms.

In A Coma And Not Expected To Wake Up Or Recover
18.If my doctor and another health care professional both decide that I am in a coma from which I am not expected to wake up or recover, and I have brain damage, and life-support treatment would only delay the moment of my death (choose one of the following): *
○ I want to have life-support treatment.

○ I do not want life-support treatment. If it has been started, I want it stopped.
○ I want to have life-support treatment if my doctor believes it could help. But I want my doctor to stop giving me life-support treatment if it is not helping my health condition or symptoms.

Permanent And Severe Brain Damage

19.If my doctor and another health care professional both decide that I have permanent and severe brain damage and I am not expected to get better, and life-support treatment would only delay the moment of my death (choose one of the following): *
○ I want to have life-support treatment.
○ I do not want life-support treatment. If it has been started, I want it stopped.
○ I want to have life-support treatment if my doctor believes it could help. But I want my doctor to stop giving me life-support treatment if it is not helping my health condition or symptoms.

Wish 3 My Wish For How I Want People To Treat Me.

20.I want them to know that these spiritual and emotional wishes are important to me, too. I wish…* (multiple-choice question)
□ I wish I can be forgiven when I behave maliciously, hurtfully, or indecently toward those around me in my sickness or old age.
□ I want to be accompanied by someone as much as possible, even though I may not be able to see, hear or feel.
□ I wish to have pictures of my loved ones in my room, near my bed.
□ I wish I can have voluntary services as much as possible.
□ I don't want to be disturbed by volunteers at any time.
□ I want to die in my home, if that can be done.
□ I wish to have my favorite music played whenpossible until my time of death.
□ I wish to have religious services specified by myself at the end of my life.
□ I don't want any religious services at any time.
□ I wish to settle or complete unfinished business.
□ Others ____________

Wish 4 My Wish For What I Want My Loved Ones To Know.

21.I wish my family and friends to treat my death calmly. Dying is a life process and natural law that everyone must go through. *（multiple-choice question）

□ I wish my family and friends to know that I love them till death.

□ I wish my family and friends can return to their normal lives as soon as possible after my death.

□ I wish my funeral will be simple.

□ I wish there won't be a memorial service for me after my death.

□ I wish that only my family and close friends will be notified of my memorial service.

□ I wish I can inform my family of their names and contact information in advance.

□ Others ____________

Wish 5 The Person I Want To Help Me

22.The person I want to make health care decisions for me When I can't make them for myself： *

○ Doctors

○ Myself (I will express my medical wishes in advance and wish that decisions will be made in accordance with my wishes expressed in advance.)

○ Families

○ Others ____________

Part Ⅳ

23. Are you willing to sign a living will "My Five Wishes" document to make medical decisions in advance so that when you are not able to make them for yourself in the future, you can provide a direction for your family and medical staff to obtain medical care that meets your wishes. *

○ Yes

○ No

(*= required)
